# Laughing

**Published:** 1874-06

**Authors:** 


### LAUGHING.

AN active circulation of the blood averts disease. It is when we are asleep, or weary, or dispirited, that the pulse is slow, making the body peculiarly susceptible to disease; but laughing increases the circulation of the blood, and as the blood is the life of a man, it carries with it new elements of vigor and deposits them all along its path. The act of laughing is preceded by a partial emptying of the blood vessels; this leaves the brain less oppressed, containing a less amount of blood, the excess going to the lungs, room for which excess is made by the forcible expulsion of air from the lungs in the act of laughing, but when we have used all the air in a laugh, the lungs instinctively demand more air; hence, we draw in a full breath and another laugh comes. While the size of the blood vessels is diminished, the size, power and activity of the nerves is increased; hence, there is more life and animation; thus laughing people are lively people; but as the nervous energy is expended in operations of he lungs, there is not much left for the brain; hence, there may be more meant than is commonly supposed in the expression, "Fat as a Fool." Some persons have laughed themselves to death from inability to take breath, or from cramps taking place from excessive exercise of the muscles employed, as in drowning from cramp; on this account some of the very best swimmers have lost their lives. If persons would strike out slowly and deliberately in swimming the dreadful and fatal cramp might be altogether avoided, especially if, when somewhat tired, a little rest should be taken by "floating."

